# Altered Ovarian Inositol Ratios May Account for Pathological Steroidogenesis in PCOS

**DOI:** 10.3390/ijms21197157

**Published:** 2020-09-28

**Authors:** Vittorio Unfer, Simona Dinicola, Antonio Simone Laganà, Mariano Bizzarri

**Affiliations:** 1 Department of Experimental Medicine, Sapienza University of Rome, Systems Biology Group Lab, 00161 Rome, Italy; simonadinicola.sd@gmail.com (S.D.); mariano.bizzarri@uniroma1.it (M.B.); 2 Department of Obstetrics and Gynecology, “Filippo Del Ponte” Hospital, University of Insubria, 21100 Varese, Italy; antoniosimone.lagana@uninsubria.it

**Keywords:** PCOS, myo-inositol, D-chiro-inositol, steroidogenesis

## Abstract

The presence of abnormal ovarian ratios of myo-inositol (MI) to D-chiro-inositol (DCI) is a recurrent feature in PCOS. Available evidence suggests that MI and DCI may modulate steroid biosynthesis, likely in an opposite manner. Specifically, MI seems to induce estrogen production, while DCI has a role in the synthesis of androgens. Elevated insulin levels, generally associated with PCOS, alter the physiological MI/DCI ratio, increasing MI-to-DCI conversion through activation of a specific epimerase enzyme. DCI directly increases testosterone biosynthesis in thecal cells and reduces its conversion to estradiol by downregulating aromatase enzyme in granulosa cells. This manuscript reviews the literature that supports the connection between altered MI/DCI ratios and pathological steroidogenesis observed in PCOS women. Furthermore, it discusses the application of inositol-based treatment protocols in managing PCOS symptoms and improving the quality of patients’ life.

## 1. Introduction

Polycystic Ovary Syndrome (PCOS) is a complex endocrine condition affecting 15-20% of women in the reproductive age [1].

The Rotterdam workshop consensus [2] established the diagnostic criteria for PCOS. Accordingly, the diagnosis is based on a combination of at least two of the following three clinical features: hyperandrogenism (clinical and/or biochemical), chronic oligo-anovulation, and polycystic ovaries at ultrasound examination.

A typical PCOS phenotype is characterized by hyperandrogenism and ovarian dysfunctions; however, other frequent abnormalities, usually not included among the canonical diagnostic criteria, may be present [3]. In particular, insulin resistance, with compensatory hyperinsulinemia, is frequently detected in several PCOS women [4].

This metabolic abnormality is present in a large proportion of overweight and obese women, but exaggerated circulating insulin levels and reduced insulin-mediated glucose metabolism were also observed in up to 40% of non-obese PCOS women [5].

Certainly, insulin is highly involved in PCOS pathogenesis, either directly or indirectly. Insulin directly prompts ovarian theca cells to enhance the synthesis and the release of androgens; in fact, elevated circulating androgen levels have been observed in 80–90% of PCOS women with oligomenorrhea [6]. Moreover, in a subgroup of PCOS women, increased insulin triggers LH receptor expression on granulosa cells of a subpopulation of small follicles, leading to premature terminal differentiation and arrest of follicular growth that may lead to anovulation [7].

In addition, high glucose concentration inhibits the hepatic synthesis of sex hormone-binding globulin (SHBG), with a consequent increase of bioavailable circulating free-androgens [8,9].

The ascertained effectiveness of insulin-sensitizing drugs, such as metformin and thiazolidinediones, in improving ovulatory function and reducing androgen excess in PCOS patients provided additional evidence that supports the pathogenic role of insulin resistance in PCOS [10]. However, patients’ compliance often suffers from side effects such as nausea and diarrhea for metformin, and increased body weight for pioglitazone. Therefore, novel effective therapeutic options, free of side effects, are highly desirable.

## 2. The Rationale of the Use of Inositol for PCOS Treatment

During the last decades, compelling evidence confirmed inositol supplementation as a pivotal and well tolerated integrative treatment for PCOS-affected women [11,12].

Inositols are cyclic polyols (C_6_H_12_O_6_) present in all living beings, where they participate to several metabolic pathways. Among the natural stereoisomers, myo-inositol (MI) is prevalent in animals [13], as natural constituent of their diet [14,15]. In humans, a number of organs (kidneys, liver, testes, mammary gland and brain) actively synthesize MI, isomerizing glucose-6-phosphate (G6P) to inositol-3-phosphate (Ins3P) [16]. Then, inositol monophosphatase-1 (IMPA-1 or IMPase) dephosphorylates Ins3P to free MI [17]. Free inositol can also be obtained by dephosphorylation of inositol-1,4,5-trisphosphate (InsP3) and inositol-bisphosphate (InsP2).

Under insulin stimulation, a specific Nicotinamide Adenine Dinucleotide (NAD)-NADH-dependent epimerase unidirectionally converts MI to d-chiro-inositol (DCI) another notable stereoisomer [18,19] according to tissue requirement.

While for most tissues the intracellular pool of inositol is almost exclusively (>99%) constituted by MI, the content of MI and DCI is significantly different in fat, muscle and liver, reflecting the distinct functions of the two isomers in those tissues [20].

Inositols participate in insulin signaling. Indeed, insulin needs the presence of both MI and DCI to exert its activity [21]. These two stereoisomers, as inositolphosphoglycans (MI-IPG and DCI-IPG), take part in the intracellular processes that control the oxidative and non-oxidative metabolism of glucose, as well as the uptake of glucose from the extracellular environment [14,15,22,23,24]. Supplementation with both MI and DCI may exert an insulin-sensitizing effect and lead to reduced insulin levels in the blood of resistant patients [25].

MI mainly controls cellular glucose uptake, and its content is significantly high in tissues with high-glucose utilization, namely the brain, the heart and the ovaries [21,24,25,26]. Insulin signal activates glucose transporters through the inositol pathway, allowing glucose to enter the cells. Moreover, dietary MI significantly prevents glucose absorption from duodenal tract and decreases glucose rise in the blood, by interfering with glucose intestinal uptake [27]. Furthermore, MI improves insulin sensitivity in adipocytes by increasing lipid storage and glucose uptake, and by inhibiting lipolysis [28].

A chief MI metabolite, -InsP3-, acts as second messenger of follicle stimulating hormone (FSH) in the granulosa cells of the ovaries [29], representing a key mediator for the selection of the dominant follicle [30]. In agreement with this physiological role, MI probably enhances serum levels of anti-Müllerian hormone (AMH), since AMH in women is produced by granulosa cells under FSH stimulation [31]. Also, in mouse models, it has been observed that MI induces the meiotic progression of oocytes into fertilization-competent eggs, whereas its reduction within the ovaries impairs physiological oocyte maturation [32].

DCI concentration is higher in tissues that store glucose as glycogen, such as liver and muscle [33].

At the ovarian level, DCI mediates insulin-induced testosterone biosynthesis from thecal cells [34], while it acts directly on steroidogenic enzymes gene regulation in granulosa cells, reducing mRNA expression of both aromatase CYP19A1 and cytochrome P450 side-chain cleavage (*P450scc*) genes in a dose-response manner [35].

Despite their chemical similarities MI and DCI in most cases exert different functions, and we could speculate that MI can affect aromatase activity in an opposite manner with respect to DCI. However experimental data in this regard are unavailable at the moment. In this perspective, higher MI/DCI ratios should increase the activity of aromatase in granulosa, inducing estrogen biosynthesis, meanwhile lower MI/DCI ratios stimulate androgen production in thecal cells [36]. Such hypothesis is based on the role played by MI (in the form of InsP3) as second messenger of FSH which is regarded as the major inducer of aromatase activity in granulosa cells [37].

For its modulatory activity on aromatase, DCI supplementation produces a systemic increase in testosterone levels, leading to a concomitant reduction of estrogens, effect increased by the direct action of DCI on testosterone synthesis in theca cells. Understandably, high levels of this isomer can exert harmful effects on oocyte quality [38,39]. On the contrary, MI reaches concentrations in the mammalian female reproductive tract significantly higher than those reported in blood serum, suggesting that it plays specific roles in the ovaries by ensuring correct oocyte maturation and transport through the oviducts [40,41].

## 3. MI to DCI Ratio Imbalance in PCOS

The presence of a specific ovarian epimerase that converts MI into DCI, indicates that both are essential for ovarian physiology and that DCI concentration is tightly regulated.

Indeed, epimerase activity is tissue specific [20], with different MI/DCI ratios in different tissues and organs. For example, the ratio is around 20:1 in the thecal cells [18] and very close to 100:1 in the follicular fluid [42].

In pathologic conditions, such as type 2 diabetes, decreased insulin sensitivity in many tissues leads to reduced epimerase activity and, consequently, lower DCI production [20,43]. Unlike most tissues, the ovaries maintain the normal insulin sensitivity, despite the presence of systemic resistance. In fact, the ovaries never become insulin resistant [31,42], and as a consequence, systemic hyperinsulinemia overstimulates epimerase activity in those tissues, causing excessive DCI synthesis at the expense of MI concentration.

Increased DCI concentration promotes androgen synthesis, while depletion of MI worsens the energy state of the oocytes, leading to impaired FSH signaling and oocyte quality. Altered ovarian MI/DCI ratios may explain the pathogenesis of PCOS in insulin resistant patients.

Evidence in support of such theory was provided by two independent studies. The first, published by Larner’s research group, analyzed the epimerase activity and the content of MI and DCI in PCOS theca cells [18]. The second, by Unfer et al., investigated the concentration of MI and DCI in the follicular fluid of healthy women and those with PCOS [42].

Both studies obtained comparable results, namely, the ovaries of healthy women presented higher concentrations of MI and lower concentrations of DCI; whereas, the ovary of PCOS patients showed a marked MI depletion and an increased DCI content.

## 4. Inositol-Based PCOS Managing: The 40:1 MI/DCI Formula

Nestler et al. in 1999 [44] observed that in obese PCOS women 1200 mg/day of DCI reduced serum testosterone level and improved ovulation rate as well as metabolic parameters, such as blood pressure and triglycerides. A further study, involving a larger number of patients and increasing the DCI dosage up to 2400 mg/day [45], was unable to confirm the results published previously. With the higher dose of DCI testosterone levels failed to decrease.

The ovarian paradox may help to understand why a supplementation with DCI alone cannot be considered a reliable approach to manage PCOS. Hence, supplementing DCI alone is not a recommendable choice for several reasons: (1) high doses of DCI have been considered toxic to ovaries and oocyte maturation; (2) DCI is not converted into MI, and thus the specific action exerted by MI would be lost; (3) MI and MI-IPG deficiencies are correlated with many insulin resistance conditions.

Classical insulin sensitizers ameliorate the metabolic and reproductive PCOS features, but side effects often lead to poor compliance. Scientific evidence demonstrated that also MI is effective in managing PCOS symptoms, and a recent meta-analysis compared the short-term effects of metformin and MI in PCOS affected women [46].

The authors demonstrated that in these patients there is no difference in the short-term effect of metformin vs MI regarding fasting insulin, Homeostasis Model Assessment (HOMA) index, testosterone, androstenedione and Sex Hormone Binding Globulin (SHBG). However, a statistically significant heterogeneity was observed for HOMA, SHBG and BMI changes. The meta-analysis confirmed that MI is associated with a lower risk of adverse events in comparison to metformin, and for this reason its use could be safer or possible also in association with lower levels of metformin in subjects that do not tolerate higher therapeutic dose of this insulin sensitizer.

The most relevant clinical results have been obtained with the combination of MI and DCI in the 40:1 ratio, which is similar to the ratio found in the plasma of healthy women [47].

Another recent meta-analysis [48] examined 9 randomized controlled trials (RCTs) on PCOS women (247 cases and 249 controls) [49,50,51,52,53,54,55,56]. The authors evaluated the efficacy of supplementing MI alone, or in association with DCI in the 40:1 ratio, considering fasting insulin concentrations as the primary outcome, while HOMA index and serum levels of testosterone, androstenedione and sex hormone-binding globulin (SHBG) as secondary. The authors reported that inositol supplementation significantly reduced fasting insulin and HOMA index, with a slight trend towards testosterone decrease with respect to controls. Moreover, a significant increase in SHBG levels was observed after MI administration.

These results were further confirmed by a systematic review and meta-analysis [57], including 10 RCTs involving a total of 573 patients. Total testosterone, estradiol (E2), and HOMA index were the primary endpoints. Compared with the control group, inositol administration significantly improved HOMA index and raised E2 levels, showing only a trend in reducing total testosterone levels.

The first clinical study comparing the results obtained from PCOS patients after the administration of different MI/DCI ratios (0:1; 1:3.5; 2.5:1; 5:1; 20:1; 40:1 and 80:1) was reported by Nordio and colleagues [58]. As primary outcome, the authors investigated the ovulation by means of progesterone assay, while as secondary outcomes they observed the improvement of the following metabolic parameters: FSH, LH, SHBG, E2, free testosterone, HOMA index, basal and postprandial insulin.

From all the ratios tested, the 40:1 yielded the best improvements, followed by the 20:1 and 80:1; instead, the other combinations showed less relevant outcomes [58].

These clinical findings perfectly agree with the results obtained by Bevilacqua et al. using a mouse model of PCOS [59]. The authors induced the syndrome by exposing 30-days-old females to 10 weeks of continuous light. They observed that the ovaries of these mice showed lack of tertiary follicles and corpora lutea, altered ovarian architecture, and increased thickness of the theca layer. PCOS signs and symptoms completely disappeared with daily supplementation of 420 mg/kg MI/DCI in a 40:1 ratio, in a more effective way than the other ratios examined. Moreover, the 40:1 formula restored the low theca/granulosa cell layer thickness values, leading to a faster recovery of murine fertility, with a physiological delivery time after mating.

The other tested MI/DCI ratios were less effective or even exerted negative effects on the clinical conditions. In particular, the formula with higher DCI content demonstrated to worsen PCOS pathological features.

Overall, both these studies support the 40:1 MI/DCI ratio as the best treatment for PCOS patients, with the aim of restoring ovulation.

## 5. Overcoming “Inositol-Resistance”

In 30–40% of PCOS women, inositol supplementation fails to improve metabolic and hormonal parameters, or to restore ovulation [52,60,61,62]. These patients are defined “inositol-resistant”, and “inositol-resistance” refers to the therapeutic inefficacy of inositols.

It has been hypothesized that the poor or absent absorption of inositol may be responsible for the lack of therapeutic effects. Several conditions, such as obesity, chronic intestinal diseases, dysbiosis etc. are candidate to represent risk factors for developing inositol-resistance.

Since alpha-lactalbumin (alpha-LA) can enhance the passage through biological barriers, combining MI with this whey protein revealed to be an effective strategy to overcome inositol resistance. Importantly, alpha-LA and inositols are both included in the FDA list of Generally recognized as safe (GRAS) compounds (https://www.accessdata.fda.gov/scripts/cdrh/cfdocs/cfcfr/CFRSearch.cfm?fr=184.1370).

Monastra and colleagues firstly demonstrated, both in vivo and in vitro, that concomitant administration of alpha-LA ameliorated the absorption and the bioavailability of MI [63]. Indeed, the simultaneous oral administration of MI and alpha-LA led to a significantly higher plasma concentration of MI with respect to MI supplemented alone. Moreover, the authors observed an increased passage of MI in the presence of alpha-LA and a concomitant lowering of the Trans-Epithelial Electrical Resistance, indicative of the opening of the tight junctions between the Caco-2 cells, used as an in vitro model of intestinal mucosa epithelial monolayer [63].

A subsequent open and prospective study on PCOS patients treated with MI and alpha-LA clinically confirmed the efficacy of this new formulation [64]. In the first part of the study, following treatment with the sole MI, 23 out of 37 women (62%) ovulated, while 14 (38%) demonstrated to be “resistant” and did not ovulate. In a second step, these MI-resistant patients were supplemented with MI plus alpha-LA. Following the combined treatment, 12 (86%) patients ovulated. Moreover, their MI plasma levels were found to be significantly higher than the baseline. Improved hormone and lipid profiles were also recorded [64].

## 6. Conclusions

**Figure 1:**
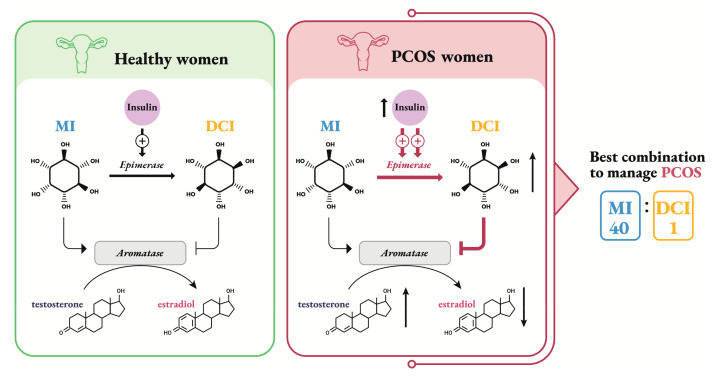
Schematic representation of how inositols affect estrogen biosynthesis in granulosa cells of healthy and PCOS women. Testosterone in granulosa cells comes from thecal biosynthesis, which is stimulated by insulin through DCI second messenger. In healthy women MI stimulates aromatase to produce estrogens; while DCI, obtained from insulin-dependent MI conversion by epimerase, has the opposite effect. In PCOS women hyperinsulinemia forces the epimerase to convert MI to DCI. This isomer has a modulating effect on aromatase, leading to a systemic increase in testosterone levels and a concomitant reduction of estrogens. A combination of MI and DCI in the 40:1 ratio seems to be the most effective to ameliorate PCOS parameters.

Inositols differently modulate the steroid biosynthesis in the ovaries: in particular, MI is likely to induce estrogen production, while DCI has a role in the synthesis of androgens (Figure 1).

In PCOS women, the connection between increased ovarian MI-to-DCI conversion and pathological steroidogenesis is often observed (Figure 1).

While supplementation with DCI may potentially induce detrimental effects on ovarian physiology, especially in PCOS women, supplementation with MI and DCI in the proper ratio (40:1) seems to represent an effective treatment for restoring ovulation in PCOS patients (Figure 1).

Moreover, since poor MI absorption in the gut may lead to therapy failure, as observed in a fraction of PCOS patients treated with inositols, the association of MI with alpha-LA allows to overcome the “inositol-resistance” and to increase the number of women who respond to MI supplementation. All these reports fruitfully enrich and enlarge the promising field of inositol studies related to PCOS, even though a validation of the concept of MI/DCI ratio and the potential role of alpha-LA with more robust studies is recommendable.

## References

[1] Barnard L., Ferriday D., Guenther N., Strauss B., Balen A.H., Dye L. (2007). Quality of life and psychological well being in polycystic ovary syndrome. Hum. Reprod..

[2] ESHRE, The Rotterdam, ASRM-Sponsored PCOS Consensus Workshop Group (2004). Revised 2003 consensus on diagnostic criteria and long-term health risks related to polycystic ovary syndrome (PCOS). Hum. Reprod..

[3] Tosi F., Bonora E., Moghetti P. (2017). Insulin resistance in a large cohort of women with polycystic ovary syndrome: A comparison between euglycaemic-hyperinsulinaemic clamp and surrogate indexes. Hum. Reprod..

[4] Dunaif A. (1997). Insulin resistance and the polycystic ovary syndrome: Mechanism and implications for pathogenesis. Endocr. Rev..

[5] Homburg R. (2008). Polycystic ovary syndrome. Best practice & research. Clin. Obstet. Gynaecol..

[6] Azziz R., Carmina E., Dewailly D., Diamanti-Kandarakis E., Escobar-Morreale H.F., Futterweit W., Janssen O.E., Legro R.S., Norman R.J., Taylor A.E. (2009). The Androgen Excess and PCOS Society criteria for the polycystic ovary syndrome: The complete task force report. Fertil. Steril..

[7] Diamanti-Kandarakis E., Dunaif A. (2012). Insulin resistance and the polycystic ovary syndrome revisited: An update on mechanisms and implications. Endocr. Rev..

[8] Teede H., Deeks A., Moran L. (2010). Polycystic ovary syndrome: A complex condition with psychological, reproductive and metabolic manifestations that impacts on health across the lifespan. BMC Med..

[9] Baillargeon J.P., Nestler J.E. (2006). Commentary: Polycystic ovary syndrome: A syndrome of ovarian hypersensitivity to insulin?. J. Clin. Endocrinol. Metab..

[10] Abdalla M.A., Deshmukh H., Atkin S., Sathyapalan T. (2020). A review of therapeutic options for managing the metabolic aspects of polycystic ovary syndrome. Ther. Adv. Endocrinol. Metab..

[11] Unfer V., Carlomagno G., Dante G., Facchinetti F. (2012). Effects of myo-inositol in women with PCOS: A systematic review of randomized controlled trials. Gynecol. Endocrinol. Off. J. Int. Soc. Gynecol. Endocrinol..

[12] Facchinetti F., Appetecchia M., Aragona C., Bevilacqua A., Bezerra Espinola M.S., Bizzarri M., D’Anna R., Dewailly D., Diamanti-Kandarakis E., Hernández Marín I. (2020). Experts’ opinion on inositols in treating polycystic ovary syndrome and non-insulin dependent diabetes mellitus: A further help for human reproduction and beyond. Expert Opin. Drug Metab. Toxicol..

[13] Michell R.H. (2011). Inositol and its derivatives: Their evolution and functions. Adv. Enzym. Regul..

[14] Bizzarri M., Fuso A., Dinicola S., Cucina A., Bevilacqua A. (2016). Pharmacodynamics and pharmacokinetics of inositol (s) in health and disease. Expert Opin. Drug Metab. Toxicol..

[15] Laganà A.S., Garzon S., Casarin J., Franchi M., Ghezzi F. (2018). Inositol in Polycystic Ovary Syndrome: Restoring Fertility through a Pathophysiology-Based Approach. Trends Endocrinol. Metab..

[16] Wong Y.H., Kalmbach S.J., Hartman B.K., Sherman W.R. (1987). Immunohistochemical staining and enzyme activity measurements show myo-inositol-1-phosphate synthase to be localized in the vasculature of brain. J. Neurochem..

[17] Loewus M.W., Loewus F.A., Brillinger G.U., Otsuka H., Floss H.G. (1980). Stereochemistry of the myo-inositol-1-phosphate synthase reaction. J. Biol. Chem..

[18] Heimark D., McAllister J., Larner J. (2014). Decreased myo-inositol to chiro-inositol (M/C) ratios and increased M/C epimerase activity in PCOS theca cells demonstrate increased insulin sensitivity compared to controls. Endocr. J..

[19] Monastra G., Unfer V., Harrath A.H., Bizzarri M. (2017). Combining treatment with myo-inositol and D-chiro-inositol (40:1) is effective in restoring ovary function and metabolic balance in PCOS patients. Gynecol. Endocrinol. Off. J. Int. Soc. Gynecol. Endocrinol..

[20] Sun T.H., Heimark D.B., Nguygen T., Nadler J.L., Larner J. (2002). Both myo-inositol to chiro-inositol epimerase activities and chiro-inositol to myo-inositol ratios are decreased in tissues of GK type 2 diabetic rats compared to Wistar controls. Biochem. Biophys. Res. Commun..

[21] Bevilacqua A., Bizzarri M. (2018). Inositols in Insulin Signaling and Glucose Metabolism. Int. J. Endocrinol..

[22] Croze M.L., Soulage C.O. (2013). Potential role and therapeutic interests of myo-inositol in metabolic diseases. Biochimie.

[23] Michell R.H. (2018). Do inositol supplements enhance phosphatidylinositol supply and thus support endoplasmic reticulum function?. Br. J. Nutr..

[24] Nestler J.E., Unfer V. (2015). Reflections on inositol (s) for PCOS therapy: Steps toward success. Gynecol. Endocrinol. Off. J. Int. Soc. Gynecol. Endocrinol..

[25] Huang L.C., Fonteles M.C., Houston D.B., Zhang C., Larner J. (1993). Chiroinositol deficiency and insulin resistance. III. Acute glycogenic and hypoglycemic effects of two inositol phosphoglycan insulin mediators in normal and streptozotocin-diabetic rats in vivo. Endocrinology.

[26] Larner J., Huang L.C., Tang G., Suzuki S., Schwartz C.F., Romero G., Roulidis Z., Zeller K., Shen T.Y., Oswald A.S. (1988). Insulin mediators: Structure and formation. Cold Spring Harb. Symp. Quant. Biol..

[27] Chukwuma C.I., Ibrahim M.A., Islam M.S. (2016). Myo-inositol inhibits intestinal glucose absorption and promotes muscle glucose uptake: A dual approach study. J. Physiol. Biochem..

[28] Kim J.N., Han S.N., Kim H.K. (2014). Phytic acid and myo-inositol support adipocyte differentiation and improve insulin sensitivity in 3T3-L1 cells. Nutr. Res. (New York N.Y.).

[29] Milewska E.M., Czyzyk A., Meczekalski B., Genazzani A.D. (2016). Inositol and human reproduction. From cellular metabolism to clinical use. Gynecol. Endocrinol. Off. J. Int. Soc. Gynecol. Endocrinol..

[30] Unfer V.B.S. (2016). Myo-inositol and dominant follicle. Int. J. Med Device Adjuv. Treat..

[31] Carlomagno G., Unfer V., Roseff S. (2011). The D-chiro-inositol paradox in the ovary. Fertil. Steril..

[32] Chiu T.T., Rogers M.S., Briton-Jones C., Haines C. (2003). Effects of myo-inositol on the in-vitro maturation and subsequent development of mouse oocytes. Hum. Reprod..

[33] Pak Y., Huang L.C., Lilley K.J., Larner J. (1992). In vivo conversion of [3H]myoinositol to [3H]chiroinositol in rat tissues. J. Biol. Chem..

[34] Nestler J.E., Jakubowicz D.J., de Vargas A.F., Brik C., Quintero N., Medina F. (1998). Insulin stimulates testosterone biosynthesis by human thecal cells from women with polycystic ovary syndrome by activating its own receptor and using inositolglycan mediators as the signal transduction system. J. Clin. Endocrinol. Metab..

[35] Sacchi S., Marinaro F., Tondelli D., Lui J., Xella S., Marsella T., Tagliasacchi D., Argento C., Tirelli A., Giulini S. (2016). Modulation of gonadotrophin induced steroidogenic enzymes in granulosa cells by d-chiroinositol. Reprod. Biol. Endocrinol..

[36] Unfer V., Forte G. (2020). Does inositol ratio orchestrate the fate of ovarian follicles?. Med. Hypotheses.

[37] Zeleznik A.J., Saxena D., Little-Ihrig L. (2003). Protein kinase B is obligatory for follicle-stimulating hormone-induced granulosa cell differentiation. Endocrinology.

[38] Ravanos K., Monastra G., Pavlidou T., Goudakou M., Prapas N. (2017). Can high levels of D-chiro-inositol in follicular fluid exert detrimental effects on blastocyst quality?. Eur. Rev. Med Pharmacol. Sci..

[39] Laganà A.S., Unfer V. (2019). D-Chiro-Inositol’s action as aromatase inhibitor: Rationale and potential clinical targets. Eur. Rev. Med Pharmacol. Sci..

[40] Chiu T.T., Rogers M.S., Law E.L., Briton-Jones C.M., Cheung L.P., Haines C.J. (2002). Follicular fluid and serum concentrations of myo-inositol in patients undergoing IVF: Relationship with oocyte quality. Hum. Reprod..

[41] Orihuela P.A., Parada-Bustamante A., Zuñiga L.M., Croxatto H.B. (2006). Inositol triphosphate participates in an oestradiol nongenomic signalling pathway involved in accelerated oviductal transport in cycling rats. J. Endocrinol..

[42] Unfer V., Carlomagno G., Papaleo E., Vailati S., Candiani M., Baillargeon J.P. (2014). Hyperinsulinemia Alters Myoinositol to d-chiroinositol Ratio in the Follicular Fluid of Patients With PCOS. Reprod. Sci..

[43] Kennington A.S., Hill C.R., Craig J., Bogardus C., Raz I., Ortmeyer H.K., Hansen B.C., Romero G., Larner J. (1990). Low urinary chiro-inositol excretion in non-insulin-dependent diabetes mellitus. N. Engl. J. Med..

[44] Nestler J.E., Jakubowicz D.J., Reamer P., Gunn R.D., Allan G. (1999). Ovulatory and metabolic effects of D-chiro-inositol in the polycystic ovary syndrome. N. Engl. J. Med..

[45] Cheang K.I., Baillargeon J.P., Essah P.A., Ostlund R.E., Apridonize T., Islam L., Nestler J.E. (2008). Insulin-stimulated release of D-chiro-inositol-containing inositolphosphoglycan mediator correlates with insulin sensitivity in women with polycystic ovary syndrome. Metab. Clin. Exp..

[46] Facchinetti F., Orru B., Grandi G., Unfer V. (2019). Short-term effects of metformin and myo-inositol in women with polycystic ovarian syndrome (PCOS): A meta-analysis of randomized clinical trials. Gynecol. Endocrinol. Off. J. Int. Soc. Gynecol. Endocrinol..

[47] Facchinetti F., Unfer V., Dewailly D., Kamenov Z.A., Diamanti-Kandarakis E., Laganà A.S., Nestler J.E., Soulage C.O. (2020). Inositols in Polycystic Ovary Syndrome: An Overview on the Advances. Trends Endocrinol. Metab..

[48] Unfer V., Facchinetti F., Orrù B., Giordani B., Nestler J. (2017). Myo-inositol effects in women with PCOS: A meta-analysis of randomized controlled trials. Endocr. Connect..

[49] Artini P.G., Di Berardino O.M., Papini F., Genazzani A.D., Simi G., Ruggiero M., Cela V. (2013). Endocrine and clinical effects of myo-inositol administration in polycystic ovary syndrome. A randomized study. Gynecol. Endocrinol. Off. J. Int. Soc. Gynecol. Endocrinol..

[50] Costantino D., Minozzi G., Minozzi E., Guaraldi C. (2009). Metabolic and hormonal effects of myo-inositol in women with polycystic ovary syndrome: A double-blind trial. Eur. Rev. Med Pharmacol. Sci..

[51] Genazzani A.D., Lanzoni C., Ricchieri F., Jasonni V.M. (2008). Myo-inositol administration positively affects hyperinsulinemia and hormonal parameters in overweight patients with polycystic ovary syndrome. Gynecol. Endocrinol. Off. J. Int. Soc. Gynecol. Endocrinol..

[52] Gerli S., Papaleo E., Ferrari A., Di Renzo G.C. (2007). Randomized, double blind placebo-controlled trial: Effects of myo-inositol on ovarian function and metabolic factors in women with PCOS. Eur. Rev. Med. Pharmacol. Sci..

[53] Pizzo A., Laganà A.S., Barbaro L. (2014). Comparison between effects of myo-inositol and D-chiro-inositol on ovarian function and metabolic factors in women with PCOS. Gynecol. Endocrinol. Off. J. Int. Soc. Gynecol. Endocrinol..

[54] Nordio M., Proietti E. (2012). The combined therapy with myo-inositol and D-chiro-inositol reduces the risk of metabolic disease in PCOS overweight patients compared to myo-inositol supplementation alone. Eur. Rev. Med Pharmacol. Sci..

[55] Benelli E., Del Ghianda S., Di Cosmo C., Tonacchera M. (2016). A Combined Therapy with Myo-Inositol and D-Chiro-Inositol Improves Endocrine Parameters and Insulin Resistance in PCOS Young Overweight Women. Int. J. Endocrinol..

[56] Pkhaladze L., Barbakadze L., Kvashilava N. (2016). Myo-Inositol in the Treatment of Teenagers Affected by PCOS. Int. J. Endocrinol..

[57] Zeng L., Yang K. (2018). Effectiveness of myoinositol for polycystic ovary syndrome: A systematic review and meta-analysis. Endocrine.

[58] Nordio M., Basciani S., Camajani E. (2019). The 40:1 myo-inositol/D-chiro-inositol plasma ratio is able to restore ovulation in PCOS patients: Comparison with other ratios. Eur. Rev. Med. Pharmacol. Sci..

[59] Bevilacqua A., Dragotto J., Giuliani A., Bizzarri M. (2019). Myo-inositol and D-chiro-inositol (40:1) reverse histological and functional features of polycystic ovary syndrome in a mouse model. J. Cell. Physiol..

[60] Iuorno M.J., Jakubowicz D.J., Baillargeon J.P., Dillon P., Gunn R.D., Allan G., Nestler J.E. (2002). Effects of d-chiro-inositol in lean women with the polycystic ovary syndrome. Endocr. Pract. Off. J. Am. Coll. Endocrinol. Am. Assoc. Clin. Endocrinol..

[61] Kamenov Z., Kolarov G., Gateva A., Carlomagno G., Genazzani A.D. (2015). Ovulation induction with myo-inositol alone and in combination with clomiphene citrate in polycystic ovarian syndrome patients with insulin resistance. Gynecol. Endocrinol. Off. J. Int. Soc. Gynecol. Endocrinol..

[62] Raffone E., Rizzo P., Benedetto V. (2010). Insulin sensitiser agents alone and in co-treatment with r-FSH for ovulation induction in PCOS women. Gynecol. Endocrinol. Off. J. Int. Soc. Gynecol. Endocrinol..

[63] Monastra G., Sambuy Y., Ferruzza S., Ferrari D., Ranaldi G. (2018). Alpha-lactalbumin Effect on Myo-inositol Intestinal Absorption: In vivo and In vitro. Curr. Drug Deliv..

[64] Montanino Oliva M., Buonomo G., Calcagno M., Unfer V. (2018). Effects of myo-inositol plus alpha-lactalbumin in myo-inositol-resistant PCOS women. J. Ovarian Res..

